# Tissue-resident memory T cells: decoding intra-organ diversity with a gut perspective

**DOI:** 10.1186/s41232-024-00333-6

**Published:** 2024-04-17

**Authors:** Mari Murakami

**Affiliations:** 1 Department of Microbiology and Immunology, Graduate School of Medicine, Osaka University, 2-2 Yamada-Oka, Suita, Osaka 565-0871 Japan; 2 Immunology Frontier Research Center, Osaka University, Osaka, 565-0871 Japan

**Keywords:** Tissue-resident memory T cells, Inflammatory bowel disease, Single-cell analysis

## Abstract

Tissue-resident memory T cells (T_RM_) serve as the frontline of host defense, playing a critical role in protection against invading pathogens. This emphasizes their role in providing rapid on-site immune responses across various organs. The physiological significance of T_RM_ is not just confined to infection control; accumulating evidence has revealed that T_RM_ also determine the pathology of diseases such as autoimmune disorders, inflammatory bowel disease, and cancer. Intensive studies on the origin, mechanisms of formation and maintenance, and physiological significance of T_RM_ have elucidated the transcriptional and functional diversity of these cells, which are often affected by local cues associated with their presence. These were further confirmed by the recent remarkable advancements of next-generation sequencing and single-cell technologies, which allow the transcriptional and phenotypic characterization of each T_RM_ subset induced in different microenvironments. This review first overviews the current knowledge of the cell fate, molecular features, transcriptional and metabolic regulation, and biological importance of T_RM_ in health and disease. Finally, this article presents a variety of recent studies on disease-associated T_RM_, particularly focusing and elaborating on the T_RM_ in the gut, which constitute the largest and most intricate immune network in the body, and their pathological relevance to gut inflammation in humans.

## Background

The most fundamental aspect of T cells is their formation of immune memory, which enables a rapid and efficient response upon reencountering foreign antigens. Among the diverse subsets of T cells, tissue-resident memory T cells (T_RM_) reside in non-lymphoid barrier tissues, placing them at the frontline of host defense and setting them apart from circulating T cells. Recent reports have demonstrated that T_RM_ are also found in circulation, raising the possibility that T_RM_ can reenter circulation, form progeny that redistribute and contribute to the circulating memory T-cell pool, and migrate back into tissues upon recall [1, 2], thereby further strengthening defense. Because of the importance of T_RM_ in host immunity, the mechanisms underlying their formation, maintenance, and function have been intensively studied, but much remains unclear. Recently, remarkable advances in next-generation sequencing and single-cell technologies have enabled us to unveil the intricacies of T_RM_ diversity in different tissues and disease scenarios, highlighting the unique features of T_RM_ induced by the microenvironmental niche [3]. Owing to their diverse functions and molecular heterogeneity, T_RM_ not only maintain and benefit host immune homeostasis and health but can also often become pathogenic. T_RM_ heterogeneity in various tissues and pathological settings is attributed to their differential dependence on transcriptional and metabolic regulators, which are induced by context-specific signals.

The intestinal mucosa faces the external environment and is constantly exposed to commensal microbes, pathogens, dietary components, and toxic antigens. Hence, it constitutes a complex and elaborate network of the immune system, in which T_RM_ play a critical role in maintaining homeostasis. Inflammatory bowel disease (IBD), namely, chronic relapsing disorders of the gastrointestinal tract, is caused by excessive immune responses in gut mucosa. T_RM_, due to their persistent localization in peripheral tissues, are more susceptible to local antigenic stimuli and tissue-intrinsic microenvironment than other T cell subsets and can elicit strong immune responses. Indeed, the involvement of a certain subset of T_RM_ in IBD has been reported, and both protective and pathogenic aspects of each T_RM_ subset have been increasingly revealed.

This review provides a comprehensive overview of T_RM_, their origins and the mechanisms underpinning their development and maintenance, and their physiological and pathophysiological relevance, highlighting their involvement in human diseases, particularly IBD.

### Fate decision of T cells—where do TRM originate from, and how are they formed?

Naïve CD4^+^ and CD8^+^ T cells undergo unique developmental programs after activation, resulting in the generation of effector and long-lived memory T cells. Memory T cells are composed of several subsets: effector memory T cells (T_EM_), central memory T cells (T_CM_), and T_RM_. In terms of localization, T_CM_ and T_EM_ recirculate throughout lymphoid and non-lymphoid organs, respectively, whereas T_RM_ reside within peripheral non-lymphoid tissues. Although contrasting hypotheses about memory T-cell differentiation have been proposed, recent studies revealing the epigenetic landscape of CD8^+^ T cells have shown that long-lived memory CD8^+^ T cells originate from a subset of effector CD8^+^ T cells that re-express genes associated with a naïve status. The open-poised chromatin at effector genes allows these long-lived memory T cells to exert effector function upon re-exposure to the antigens [4, 5].

**Fig. 1:**
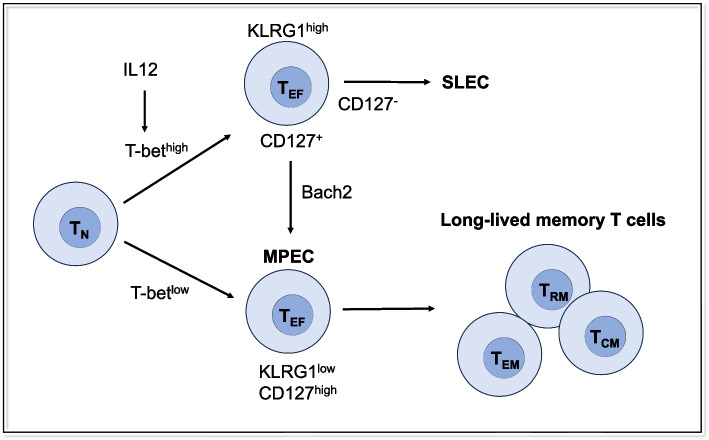
Developmental process of CD8^+^ T cell lineages. High T-bet expression, which is induced by high levels of inflammatory cytokines (i.e., IL-12), induces CD127^lo^ SLEC, while low T-bet expression promotes the induction of CD127^hi^ MPEC, which are capable of generating long-lived memory CD8^+^ T cells. KLRG1^+^ T cells receiving intermediate amounts of inflammatory signals downregulates KLRG1 in a *Bach2*-dependent manner and differentiated into all memory T cell linages. SLEC short-lived effector cells, MPEC memory precursor effector cells, KLRG1 killer cell lectin-like receptor G1, T_N_ naïve T cells, T_EF_ effector T cells, T_RM_ tissue-resident memory T cells, T_EM_ effector memory T cells, T_CM_ central memory T cells

Effector T cells (T_EF_) have been divided by the expression of CD127 and killer cell lectin-like receptor G1 (KLRG1) [6] (Fig. 1). CD127^hi^ T_EF_ highly express antiapoptotic molecules and give rise to memory cells that persist and exert long-term protective immunity. Thus, selective expression of CD127 identifies memory precursor effector cells (MPEC) [7, 8] that can give rise to both resident and circulating memory T cells [8, 9]. Long-lived circulating memory T cells are derived from KLRG1^lo^ CD127^hi^ precursor cells, whereas KLRG1^hi^ CD127^lo^ cells give rise to short-lived effector cells (SLEC) [6, 8]. Longitudinal tracking of T cells revealed the developmental plasticity of KLRG1^hi^ CD8^+^ T_EM_, which display downregulation of KLRG1 in a *Bach2*-dependent manner to efficiently differentiate into all memory T-cell lineages that are highly effective in antiviral and antitumor responses [10].

T_RM_ are transcriptionally and phenotypically distinct from T_CM_ and T_EM_ [9]_,_ making them a unique subset of memory T cells, with distinct migration patterns and localization in peripheral tissues. Resensitization of T_RM_ initiates broad local immune activation, including innate to adaptive immune systems, which leads to amplification of the local immune response to unrelated antigens [11, 12]. Indeed, it has been reported that localized skin infection generates long-lived non-recirculating CD8^+^ T_RM_ that reside throughout the skin [13]. CD103^+^ CD8^+^ T_RM_ developing in the skin and gut are derived from precursor cells that lack KLRG1 expression [14, 15] and require microenvironmental cues such as transforming growth factor β (TGF-β) and interleukin 15 (IL-15) for the formation of long-lived memory T cells and specific localization within the tissue [9, 16, 17]. TGF-β induces the expression of CD103, an αE subunit of αEβ7 integrin that interacts with E-cadherin expressed on epithelial cells, on T cells [18–21], and controls various aspects of T_RM_ development in different tissues [17, 19, 22]. TGF-β plays location- and stage-specific roles in T_RM_: in secondary lymphoid organs during the formation phase of T_RM_, TGF-β signaling to T cells inhibits the intestinal homing capacity of effector CD8^+^ T cells by inhibiting integrin α4β7 expression, which is required for T-cell trafficking to peripheral tissues [17]. During the maintenance phase, TGF-β induces integrin αEβ7, which is required for T_RM_ retention [17]. TGF-β is produced by many cell types in an inactive form and thus requires activation for bioactivity [23]. In skin epidermis, TGF-β is activated by the integrins αvβ6 and αvβ8. Regulated activation of TGF-β by these integrins expressed on keratinocytes is required for the persistence of epidermal T_RM_ [21, 24]. Type 1 regulatory T cells (Treg) promote the generation of CD8^+^ T_RM_ by making TGF-β bioavailable in the microenvironment. Mechanistically, Treg that express functional TGF-β-activating integrin αvβ8 [25] are recruited to the site of inflammation via cysteine-X-cysteine chemokine receptor 3 (CXCR3), and localized in close proximity to CD8^+^ T cells, making bioactive TGF-β locally available and promoting CD8^+^ T_RM_ development [26]. The establishment of T_RM_ depends on the presence of Treg that match the type of local infection, among which type 1 Treg are the most important population for T_RM_ development [27]. Notably, sustained TGF-β requirement for CD8^+^ T_RM_ formation depends on the tissue: it is crucial for the skin, gut and salivary gland, but not for kidney, adipose tissue, and liver [19, 28].

T_RM_ and T_CM_ share a common clonal origin despite the fact that they exhibit distinct effector properties: T_RM_ show rapid, tissue-specific responses to antigenic challenge, while T_CM_ exhibit a slower reaction [29]. Although accumulating studies have illuminated the key transcriptional regulators of T_RM_ differentiation and maintenance (see the section “Transcriptional network of T_RM_” for details), it has remained unclear whether and how certain subsets of effector T cells possess potency to commit to the T_RM_ lineage. Analyses using single-cell technology have revealed the high heterogeneity within the effector CD8^+^ T-cell population and led to intensive debate about the early precursors of T_RM_ [30–32]. A recent report suggested that a subset of circulating T_EF_ harbor a transcriptional signature similar to T_RM_ and that T_RM_-forming propensity is acquired before tissue entry [31, 33]. Additionally, DNGR-1-mediated cross-presentation by dendritic cells in draining lymph node is required for optimal T_RM_ priming [30]. Meanwhile, another study suggests that the transcriptional program of T_RM_ induced by local cues is initiated rapidly after tissue entry [32]. A recent report has shown that priming in draining lymph node initiates T_RM_ gene signatures and further license T_RM_ differentiation in response to a local factor [33]. Further studies are required to obtain a deeper understanding of the early priming of T_RM_ fate specification.

### Markers associated with TRM

T_RM_ display phenotypic variance between tissues and the environment. In general, the major hallmark of T_RM_ is the expression of CD69 and CD103. CD69 limits egress from lymphoid organs and peripheral tissues by antagonizing sphingosine-1-phosphate (S1P) receptor 1 (S1PR1) [34–36]. S1PR1 expressed on T cells senses S1P concentration gradients, which leads to the chemical migration of these cells, mediating the egress of T cells from lymphoid tissues [37]. *S1pr1* transcription is driven by Kruppel-like factor 2 (KLF2) [38], and S1P1 and KLF2 are both downregulated in T_RM_ [39]. Meanwhile, CD103 is upregulated upon exposure to TGF-β [18–21]. KLRG1 may compete with CD103 for its interaction with E-cadherin in the mucosa, and its downregulation contributes to the generation of T_RM_ [40]. Meanwhile, CD49a, the α chain of the α1β1 integrin very late antigen-1 (VLA-1), is expressed in a subset of T_RM._ CD49a-expressing T_RM_ exhibit increased effector potential compared with their CD49a-negative counterparts [41]. Upon viral infection, CD49a is important for the persistence and locomotion of virus-specific CD8^+^ T_RM_ [42, 43], demonstrating that CD49a may contribute to local surveillance of the T_RM_. Additionally, CXCR6, a receptor for C-X-C motif chemokine ligand 16 (CXCL16), is also one of the core transcriptional signatures of T_RM_ and plays a crucial role in their maintenance, localization, and function [44–47]. CXCR6 directs CD8^+^ T_RM_ homing to the airways from the lung interstitium by promoting movement within the tissue along the concentration gradient of CXCL16 [47] which is a membrane-anchored chemokine that can be cleaved by proteases to form a chemo-attractive gradient [48]. In skin, CXCR6 contributes to the process of T_RM_ formation by playing a role in local survival [46]. Elevated expression of costimulatory molecule inducible T-cell co-stimulator (ICOS) promotes the differentiation of CD8^+^ T cells into T_RM_ by enhancing the phosphoinositide 3-kinase signaling pathway, although it is not required for the maintenance of CD8^+^ T_RM_ in the tissue sites [49]. This contrasts with the requirement for ICOS to sustain long-lived CD4^+^ T follicular helper cells (Tfh) [50]. Under pathological conditions, a subset of CD103^+^ CD4^+^ T_RM_, expressing CD161 and chemokine receptor 5 (CCR5) are predominant producers of pro-inflammatory cytokines in the lamina propria of IBD, suggesting the importance of this specific T_RM_ subset in the pathogenesis [51]. Altogether, each cell surface marker associated with T_RM_ plays distinct roles in the development, function, and retention of T_RM,_ with each marker contributing to the unique phenotype and function of T_RM_ in different tissues.

T_EM_ acquire the expression of homing receptors, which are primed in draining lymph nodes to migrate to specific tissues [52]. Trafficking of T_EM_ to the skin depends on cutaneous lymphocyte-associated antigen CCR4 and CCR10, which are the receptors for C–C motif chemokine ligand 17 (CCL17) and CCL27 expressed on the skin, respectively [53, 54]. In the gut, interactions between CCR9 and its ligand CCL25, which is highly expressed in the small intestine, are required for memory T-cell homing to the small intestine, while they do not appear to be essential for T-cell migration to the colon [55, 56]. These findings illustrate that distinct factors are required for the formation and maintenance of T_RM_ depending on their microenvironment, and thus the dependence on each factor varies from tissue to tissue.

### Transcriptional regulation of TRM

**Fig. 2:**
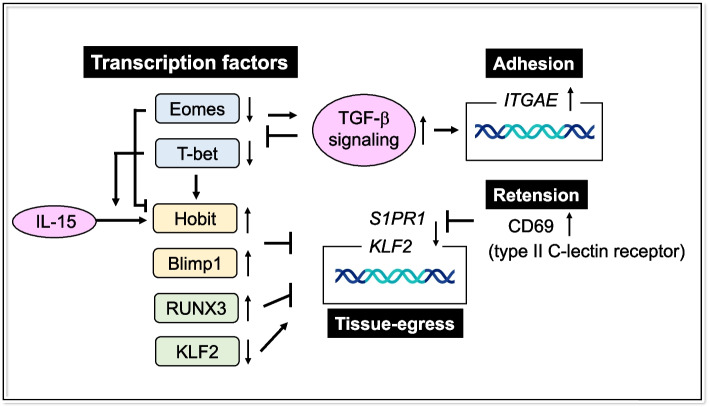
Transcriptional network of T_RM_. Hobit and Blimp1 govern the transcriptional program of T_RM_ by suppressing the expression of genes related to tissue egress. Coordinated downregulation of Eomes and T-bet is crucial for TGF-β signaling, which is required for efficient T_RM_ formation. TGF-β, in turn, downregulates Eomes and T-bet expression. TGF-β induces the expression of CD103 and controls various aspects of T_RM_ development in different tissues. CD69 limits egress from lymphoid organs and peripheral tissues by antagonizing S1PR1

Recent studies have begun to elucidate the transcriptional mechanisms underlying the differentiation, survival, maintenance, and function of T_RM_ (Fig. 2). Hobit and Blimp1 govern a transcriptional program of CD8^+^ T_RM_ by suppressing the expression of genes related to tissue egress, such as *Klf2*, *S1pr1*, and *Ccr7*, by directly binding to those genes [57]. IL-15 induces Hobit, but not Blimp1, in a T-bet-dependent manner, which in turn results in the induction of CD8^+^ T_RM_ [57]. Functional impairment of Hobit and Blimp-1 in animals was shown to attenuate colitis, as a result of impaired cross-talk between the adaptive and innate immune systems [58].

The T-box transcription factor Eomesodermin (Eomes) and its related homolog T-bet are tightly regulated during T_RM_ development [14, 59]. The lineage determination by T-bet is complex. A gradient of T-bet created in response to the amount of inflammation influences the fate of memory cells: high T-bet expression, which is induced by high levels of inflammatory cytokines (i.e., IL-12), induces CD127^lo^ SLEC, while low T-bet expression promotes the induction of CD127^hi^ MPEC, which are capable of generating long-lived memory CD8^+^ T cells [8], including CD8^+^ CD103^+^ T_RM_ [9] (Fig. 1). Coordinated downregulation of Eomes and T-bet is crucial for TGF-β signaling, which is required for efficient T_RM_ formation [14]. T-bet is capable of binding to the *Itgae* locus, which encodes CD103 [59]. Notably, a putative Smad3 binding site overlaps with the T-bet binding site, implying that T-bet suppresses T_RM_ formation by suppressing *Itgae* transcription, possibly through competing for the binding site with pSmad3, which is downstream of TGF-β signaling [59]. Meanwhile, TGF-β downregulates Eomes and T-bet expression, which in turn leads to increased TGF-β receptor signaling by a forward feedback loop [14]. Although both T-box transcription factors decline with maturation of T_RM_ and Eomes expression is lost in the final stage, a low level of residual T-bet maintains the responsiveness of T_RM_ to IL-15 [14]. A study using Hobit reporter/deleter mice showed that Hobit-expressing T_EF_ formed T_RM_ precursors and downregulated Eomes in the early phase of T_RM_ differentiation, indicating that Eomes is a key factor responsible for the early bifurcation of resident and circulating memory cell lineages [60]. Epigenetic analysis suggests that Eomes and T-bet may compete for *Hobit* locus by suppressing and inducing this gene, respectively, and dictate T_RM_ differentiation [60]. Decreased T_RM_ in the lungs of infants is attributed to elevated T-bet, implying that targeting this key molecule in infancy could promote long-term, tissue-targeted protection at this critical life stage [61].

The Runx (Runt-related transcription factor) family of transcription factors, particularly RUNX3, have been shown to be associated with various aspects of the tissue residency of T cells [62, 63]. Runx1, which is highly expressed on naïve T cells, is downregulated as the cells differentiate into T helper 1 cells (Th1), while Runx3 is upregulated [64]. Specifically, Runx3 is a key transcription factor for CD8^+^ T_RM_ differentiation and maintenance by upregulating genes associated with tissue residency while suppressing tissue egress-related genes, such as *Klf2* and *S1pr1* [62]. Noteworthy, RUNX3 expression is repressed in CD4^+^ T cells via CD4^+^ lineage-specific transcription factor ThPOK, which renders it unresponsive to TGF-β. This indicates that formation of CD8^+^ and CD4^+^ T_RM_ is regulated by distinct mechanisms [63]. In pathological settings, RUNX3 enhances CD8^+^ tumor-infiltrating lymphocytes (TIL) in melanoma, which results in tumor growth inhibition [62]. Additionally, human skin-resident CD8^+^ T_RM_ require both RUNX2 and RUNX3 for the induction of cytotoxicity and the expression of CD49a [65]. RUNX2 has been shown to promote the acquisition of a tissue-resident phenotype in natural killer cells in humans, but is not responsible for their cytotoxicity [66].

Notch signaling has been implicated in the early formation and maintenance of CD4^+^ memory T cells [67–70] and effector differentiation of CD8^+^ T cells [69, 71]. The activation of Notch involves subsequent proteolytic cleavages, and the intracellular domain of Notch then translocates to the nucleus and acts as a transcriptional regulator [69]. This signaling pathway has also been linked to the formation and maintenance of both CD4^+^ and CD8^+^ T_RM_, particularly in the context of lung T_RM_ [70, 72, 73].

Recent research has highlighted the tight association of local signals with the differentiation, homeostasis, and functions of T_RM_ [74, 75]. Site- and context-specific regulation of T_RM_, an emerging concept that plays a role in strengthening the barrier function of the unique microenvironment in each organ, is acquired by tissue-specific chromatin accessibility changes. For instance, molecular and functional heterogeneity of T_RM_ between the small intestine and colon has been attributed to the differential dependence on Eomes, which is not essential for T_RM_ formation but supports the maintenance of established T_RM_ in the small intestine [76]. However, this is not the case in the colon, highlighting the differential maintenance of these specific T_RM_ populations. Additionally, tissue-specific transcriptional regulator Hic1 is a critical regulator of T_RM_ differentiation in the small intestine by promoting the expression of P2X purinoceptor 7 which facilitates TGF-β responsiveness [28].

### Metabolic regulation of TRM

Accumulating evidence has shown that T_RM_ formation or maintenance requires distinct metabolic adaptations to different tissue environments. Reflecting the diversity of T_RM_, these cells exhibit metabolic rewiring that equips them with the ability to respond quickly to their microenvironment. T_RM_ are locked in an activated state similar to T_EF_. The controlled activation state of intraepithelial lymphocytes (IEL), a type of T_RM_ residing in the gut, has been associated with the cardiolipin composition of the mitochondrial membrane, which changes to support cell proliferation and effector function upon inflammation [77]. Indeed, the regulation of mitochondrial fitness by the transcription factor Bhlhe40 is integral to the function, development, and maintenance of T_RM_ and TIL [78]. In line with this, CD8^+^ T_RM_ exhibit increased mitochondrial oxidative metabolism in a manner dependent on the uptake of exogenous free fatty acids by fatty acid-binding protein (FABP), suggesting that oxidative metabolism is important for the tissue residency of CD8^+^ T_RM_ and their mediation of protective immunity [79, 80]. The type of FABP isoform expressed on T_RM_ is tissue-dependent and modified in line with their new location when the cells relocate to different organs [80]. Additionally, glucose availability in the local environment can regulate IEL activity, resulting in rapid pathogen clearance in the gut [81]; meanwhile, the insulin signaling pathway in intestinal T cells promotes the differentiation of T_EF_ into T_RM_ through H3K27 methylation on specific gene loci [82]. Moreover, the metabolic programs of T_RM_, which are skewed toward the sterol regulatory element-binding protein 2 (SREBP2)-dependent pathway, enhance tumor immunity, providing insights into potential therapeutic strategies that leverage the unique metabolic features of T_RM_. Interestingly, this metabolic adaptation was found to be most pronounced in the small intestine rich in dietary cholesterol [83]. Retinoic acid (RA), a vitamin A metabolite and one of the key factors in the maintenance of intestinal homeostasis, is produced by commensal bacteria in the gut [84]. Conversely, the production of RA by host intestinal epithelial cells is controlled by the gut microbiota [85]. RA enhances the expression of integrin α4β7 and CCR9 on T cells, which are essential for a preferential homing to the gut, especially to the small intestine [86]. This is mediated by RA receptor-α which binds to RA-response elements on regulatory region of the integrin α4 gene [87]. Additionally, T cell priming in mesenteric lymph nodes (MLNs) regulates CD103^+^ T_RM_ differentiation in the intestine via RA signaling [33]. Thus, RA is involved in both homing of T cells to the gut and in situ differentiation induction of T_RM_.

### *CD4*^+^*TRM and CD8*^+^*TRM*

CD8^+^ T cells are restricted by major histocompatibility complex class I (MHC-I) molecules with cytotoxic functions, whereas CD4^+^ T cells are MHC-II-restricted and programmed for helper functions, triggering the immune response by recognizing pathogens and secreting cytokines. CD8^+^ T_RM_ serve as local sensors, initiating proliferation in response to local antigen stimulation, and functioning as frontline alarm systems at sites of microbial exposure [88]. In contrast to CD8^+^ T_RM_ that reside in tissue for long periods and play a critical role in local immunosurveillance, the roles of their CD4^+^ counterparts have been less clearly described. This is despite the greater abundance of CD4^+^ T cells throughout the body except for the intestine, where CD4^+^ and CD8^+^ T cells are comparable in number [89]. A previous report demonstrated that CD4^+^ T_RM_ are superior to circulating memory T cells in terms of mediating protection against viral infection [90]. CD4^+^ T_RM,_ as well as CD8^+^ T_RM,_ remain within non-lymphoid tissue, sharing their core transcriptional signatures with those of their CD8^+^ counterparts, which are characterized by the upregulated expression of certain markers such as CD69 and CD103 [45, 91]. Meanwhile, there are differences between CD8^+^ T_RM_ and CD4^+^ T_RM_; CD8^+^ and CD4^+^ T_RM_ differ in tissue residency that is attributed to lack of TGF-β responsiveness in CD4^+^ T_RM_, resulting from divergent RUNX3 activity [63]. CD8^+^ T_RM_ possess cytolytic functions by the production of interferon gamma (IFN- γ) and tumor necrosis factor alpha (TNF-α). Meanwhile, CD4^+^ T cells in the skin exhibit a more dynamic pattern of migration and recirculation than cutaneous CD8^+^ T cells that reside in the epidermis and are confined largely to the original site of infection [92]. Mechanistically, CD4^+^ CD69^+^ CD103^+^ T_RM_ in skin downregulate CD69 and exit the tissue. Indeed, a skin-tropic CD4^+^ CD69^−^ CD103^+^ population is found in lymph and blood that is clonally related to the CD4^+^ CD69^+^ CD103^+^ T_RM_ in skin [1]. Additionally, CD4^+^ T_RM_ are inherently less proliferative and the molecular mechanisms underlying the generation of memory CD4^+^ T cells remain elusive [91]. Examination of the turnover of CD4^+^ T cells in transplanted duodenum in humans revealed that the majority of CD4^+^ T cells are donor-derived even a year after transplantation and that the vast majority of intestinal CD4^+^ T_RM_ are polyfunctional T cells with a Th1-skewed phenotype [93], similar to CD4^+^ T_RM_ observed in inflamed human gut [51] and human lung [73]. Lung CD4^+^ T_RM_ discretely remodel epithelial cell responses during heterotypic memory-recall infection, which enhance the stability of the CXCL5 transcript via IL-17A and thus accelerate neutrophil recruitment to the lung [94]. Additionally, T helper cells that exhibit both Tfh and T_RM_ features provide local assistance for the optimal development of tissue-resident memory B and CD8^+^ T cells after viral infection, uncovering the presence of a subset of T_RM_ in the lung that play a critical role in promoting the development of protective B-cell and CD8^+^ T-cell responses [95, 96]. The cytokine milieu is also involved in the formation, residency, and maintenance of CD4^+^ T_RM_. IL-2 signaling, which is important for CD4^+^ T-cell regulation and generation of memory [97], is required for tissue residency in lung allergy-driving CD4^+^ Th2 T_RM_ and the maintenance of viral antigen-specific CD4^+^ Th1 T_RM_ in the lung [98, 99]. A further overview of CD4^+^ T_RM_ can be obtained by referring to another review article [100].

### The role of TRM in health and disease — protective and pathogenic aspects of TRM

Since T_RM_ reside in non-lymphoid organs in the periphery and are locked into the effector-poised state, as indicated by the different transcriptional profiles from circulating T_EM_ and T_CM_, T_RM_ can respond rapidly and serve as the frontline of host defense against invading pathogens. Containment and rapid elimination of invading pathogens by T_RM_ at the site of entry are beneficial to the host, avoiding tissue damage and systemic dissemination. Indeed, numerous studies have demonstrated the critical roles of T_RM_ against microbial infection such as by herpes simplex virus 2, leishmania, bacterial pathogens, and viruses, in the vagina, lungs, and other mucosal sites [90, 101–104], and both CD8^+^ T_RM_ and CD4^+^ T_RM_ have been shown to contribute to this process [59]. For example, in the case of CD4^+^ T_RM,_ skin CD4^+^ T_RM_ enhance protection against leishmania through the production of IFN-γ by pathogen-specific T_RM_ and the recruitment of circulating T cells to skin in a CXCR3-dependent manner [101]. Another study demonstrated that parenteral immunization can lead to CD4^+^ T_RM_ generation in nasal tissue, playing a crucial role in defending against pneumococcal infection [103]. Taken together, these findings highlight the significance of T_RM_ as a key component of immune responses to microbial threats, particularly in the context of local infection. Barrier tissues harbor diverse commensal microorganisms, such as bacteria and fungi. Interactions between commensal microbes and the host immune system, particularly in the gut, can lead to the generation of T cells, which are reactive to the microbiome. Microbiota-reactive T_RM_ are abundant in the gut of healthy individuals and might play a significant role in supporting gut homeostasis by producing barrier-protective cytokines and providing a large pool of T cells with potential reactivity toward newly encountered pathogens [105]. During inflammation, functions of microbiota-reactive T cells can be altered: in patients with IBD, microbiota-reactive tissue-resident CD4^+^ T cells exhibit a Th17-skewed phenotype, possibly reflecting the protective effect of the host to boost tissue integrity [105]. Additionally, T_RM_ take part in local cancer immunosurveillance and are associated with a better response to cancer treatments. T_RM_ express inflammatory cytokines, cytolytic proteins, and immune checkpoint molecules, indicating their antitumor role within the tumor [106–108]. Further details can be obtained by referring to various review articles on the action of T_RM_ in cancer [109–111].

Excessive responses of T cells can lead to inflammation and tissue damage, indicating the importance of maintaining an appropriate balance between pathogen elimination and immunopathology. In contrast to the protective aspect of T_RM_, pathogenic phenotypes of T_RM_ have also been implicated in various diseases, including autoimmune disorders, such as vitiligo, psoriasis, and cutaneous lupus [112]. In organ transplantation, the donor T cells persist for a long time, whereas lung-infiltrating T cells gradually acquire T_RM_-like phenotypes. As a result, persistence of donor T cells in the recipient is associated with clinical complications after lung transplantation [113]. The infiltration of donor CD8^+^ T_RM_ into the recipient’s gastrointestinal tract has also been attributed to gastrointestinal acute graft-versus-host disease [114].

### The role of TRM in the regulation of gut inflammation

The intestinal tract is constantly exposed to foreign antigens such as microorganisms and dietary components. Although the antigens associated with IBD have not been fully elucidated, such antigens induce localized recurrent inflammation [105, 115]. Thus, it is reasonable to assume that the immunological recall function of the T_RM_ and their ability to activate local immune responses is involved in the pathogenesis of IBD. Indeed, T_RM_ have been implicated in IBD, with conflicting results having been obtained in various studies. This can be partially due to T_RM_ heterogeneity, in addition to the high variance of the patient cohorts between the studies. IBD is a chronic, relapsing, and inflammatory disorders of gastrointestinal tract, which consists of Crohn’s disease (CD) and ulcerative colitis (UC). It has been conventionally proposed that CD has been associated with Th1 and Th17, whereas UC with Th2 and Th17, suggesting the involvement of cytokines in the pathogenesis of IBD. Studies using a mouse model of colitis have suggested that T_RM_ have a pathogenic effect in this condition. Double deficiency of T_RM_-associated transcription factors Hobit and Blimp1 in T cells protected mice from various colitis models, indicating the essential role of the T_RM_ subset in the development of colitis [58]. In these knockout (KO) mice, cross-talk between the adaptive and innate immune systems was impaired, leading to protection against colitis development [58]. Another study in mice revealed that insulin receptor expressed on gut T cells promotes T_RM_ differentiation, especially for CD4^+^ T_RM_, via enhancer of zeste homolog 2 (EZH2), and exacerbates intestinal inflammation by promoting the secretion of cytokines such as TNF and IL-17 [82]. Interestingly, in a mouse model of prodromal Parkinson’s disease that develops enteritis with loss of enteric neurons, Th1/17 CD4^+^ T_RM_ are also activated in the gut mucosa during inflammation. Notably, depletion of CD4^+^ T cells partially restores enteric neurodegeneration in these mice [116]. Despite a variety of experimental animal models of enteritis, such as IL-10 KO, T-cell transfer into recombination activating gene KO, and dextran sulfate sodium-induced colitis, which are widely used to study the molecular mechanisms underpinning IBD and can indeed partially replicate certain aspects of the disease, none of them fully reproduces the complex pathophysiology of human IBD. This is because IBD is a complex, multifactorial disease involving genetic and environmental factors. Furthermore, there are substantial differences between the human immune system and that of mice.

**Fig. 3:**
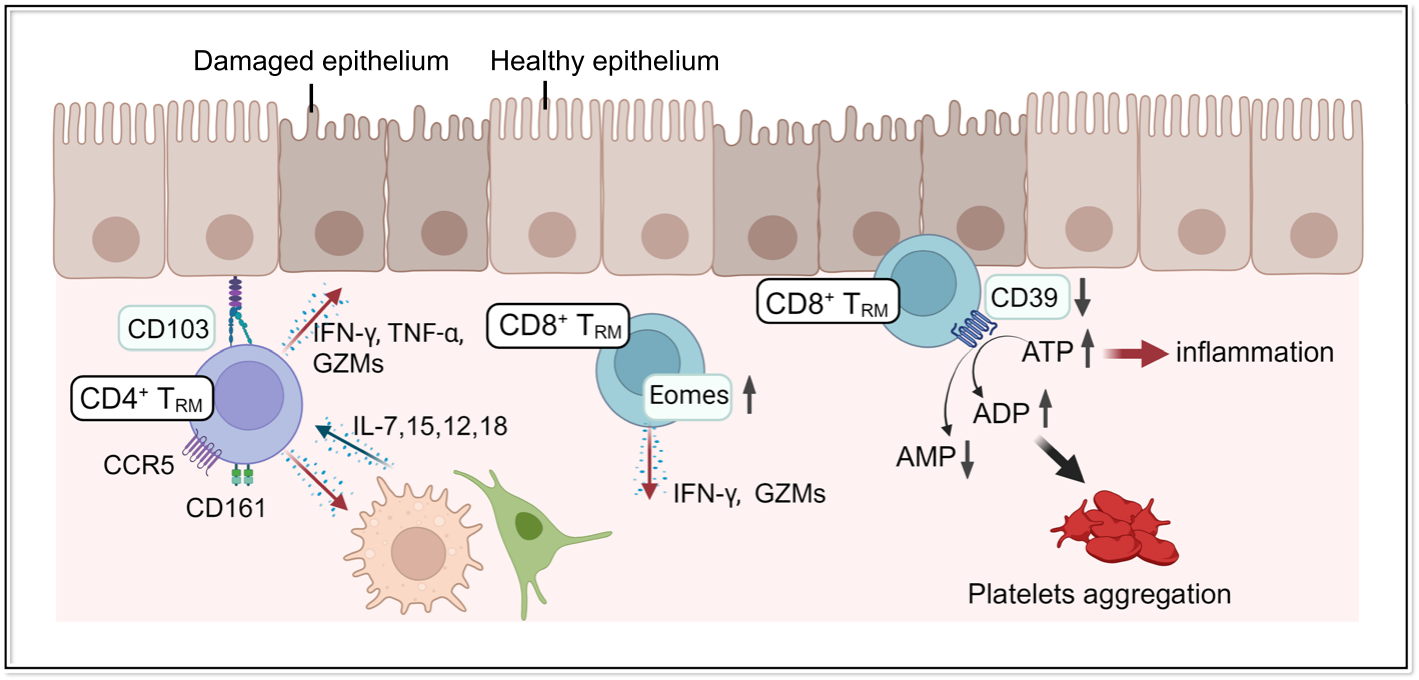
T_RM_ in IBD. In IBD, certain subsets of T_RM_ have been implicated in the pathogenesis of IBD. A subset of CD4^+^ CD103^+^ T_RM_, which is increased in Crohn’s disease, becomes activated by cytokines that are abundant in the gut mucosa of IBD patients. These T_RM_ secrete inflammatory cytokines and cytotoxic granules, contributing to the induction of inflammation. In UC, CD8^+^ T_RM_ with high Eomes expression undergo clonal expansion in the gut mucosa and express high levels of inflammatory cytokines, chemokines, and cytotoxic granules. In contrast, CD39-expressing CD8.^+^ T_RM_ are reduced in IBD, leading to inflammation triggered by the accumulation of ATP and ADP (figure created by BioRender)

Recent reports have shed light on T_RM_ as one of the important hallmarks of gut immunity in patients with IBD (Fig. 3). Indeed, the number of T_RM_ is altered in the gut mucosa of IBD patients compared to the healthy gut. CD4^+^ T_RM_ are expanded in the gut specimens of patients with CD [51, 117, 118], UC [119], or both [58], while another report revealed a decreased proportion of CD4^+^ T_RM_ [120]. The proportion of CD8^+^ T_RM_ is decreased in UC [121], or in both subtypes of IBD [120, 122–124], while a certain subset of CD8^+^ T_RM_ is expanded [125]. Previous reports have mostly suggested that certain subsets of CD4^+^ T_RM_ might be pro-inflammatory, whereas an altered population of CD8^+^ T_RM_ might be immunosuppressive in the gut of IBD patients. However, a specific part of the CD8^+^ T_RM_ fraction expressing Eomes and clonally expanded in UC is actually pro-inflammatory, exhibiting enhanced inflammatory properties [125]. It is intriguing that Eomes, whose expression is downregulated to enable responsiveness to TGF-β for T_RM_ differentiation, may be a crucial molecular regulator of a pathogenic CD8^+^ T_RM_ in UC. In immune checkpoint inhibitor (ICI)-colitis, CD8^+^ T_RM_ are the dominant activated T-cell subset that correlates with clinical and endoscopic ICI-colitis severity [126]. Expanded CD4^+^ T_RM_ are a major source of Th1 and Th17 cytokines in CD [51, 117] and UC [119], although a study has indicated that inflammatory T_RM_ are rarely expressed in UC, in contrast to the case in CD [51]. Notably, CD-specific CD4^+^ T_RM_ display an effector and innate-like nature characterized by exogenous T-cell receptor-independent activation to promote the secretion of cytolytic molecules and proinflammatory cytokines [51]. An important aspect of T_RM_ is that their long-term presence in non-lymphoid peripheral tissues allows them to exhibit context-specific functions through reprogramming under the influence of local cues. This is in contrast to T_EM_ and T_CM,_ which recirculate between blood and peripheral or lymphoid organs, respectively. For instance, CD-specific CD4^+^ T_RM_ are poised for the rapid execution of effector functions upon activation by IL-7, IL-12, IL-15, and IL-18, all of which have been shown to be abundant in the lamina propria of IBD gut [51]. This indicates that the unique microenvironment can further enhance the functional properties of these cells. Furthermore, CD4^+^ T_RM_ in the lamina propria as well as IEL reside in close proximity to the gut epithelia, and this spatial property of T_RM_ may further exacerbate epithelial injury [51, 58]. The proportion of CD4^+^ T_RM_ in the affected lamina propria is associated with the clinical status, such as having positive and negative correlations with the clinical score and flare-free survival, respectively [51, 58]. It is possible that the T_RM_ that were found to be reduced in acute IBD [120] are T cells with regulatory functions induced by contact with intestinal epithelial cells [127], although the heterogeneity of T_RM_ has not been elucidated in this study.

Analysis of the gut mucosa of IBD patients revealed decreased CD39-expressing CD8^+^ T_RM_ in patients with IBD [123, 124]. Another study also showed a decrease in global CD8^+^ IEL including a subset of CD39^+^ CD103^+^ CD8^+^ T cells [118]. CD39, encoded by *ENTPD1*, degrades excessive extracellular adenosine triphosphate (ATP) and adenosine diphosphate (ADP) into adenosine monophosphate. Since ADP is a platelet agonist, the increase in ADP associated with the reduction of CD39-expressing CD8^+^ T_RM_ may also be involved in platelet aggregation and exacerbate inflammation [123]. Additionally, extracellular ATP and ADP in the gut have been found to play a role in promoting colitis [128]. Together with the finding that regulatory T-cell function is mediated by CD39 [124], decrease of CD39-expressing CD8^+^ T_RM_ may exacerbate colonic inflammation [123, 124]. The complexity of these CD39-expressing CD8^+^ T_RM_ in the context of IBD is that this subset simultaneously expresses a transcriptional signature with a cytolytic or effector status (i.e., *GZMs*, *IFNG*) [118, 123] and regulatory molecules (i.e., *LAG3*, *TIGIT*) [123], implying that this subset is equipped with opposing regulatory networks. Another study involving comprehensive analysis of gut immune cells in UC patients revealed transcriptionally distinct subsets within CD8^+^ T_RM_. One of these subsets, which exerts enhanced effector and cytolytic properties governed by the transcription factor Eomes, was clonally expanded [125]. Interestingly, clonally related T cells in the peripheral blood were also increased, which may reflect that this T-cell fraction exits the gut and recirculates, as recently described [1, 2].

## Conclusion

Tissue-resident immune cells, especially those in the gastrointestinal tract and skin that are in close contact with the external environment, are more susceptible to local environmental factors than circulating T cells, and undergo unique adaptations. Molecular and functional diversity of T_RM_, depending on their state of equilibrium with other immune cells, can lead to various phenotypes in the host, often having protective or detrimental effects. Much remains to be understood about tissue- or context-specific cues that drive and specify the function of a certain subset of T_RM_. In particular, the investigation of T_RM_ in humans is important, despite many challenges in human tissue sampling, and the differences in immune systems between species when extrapolating the findings of animal experiments to human physiology should also be considered. Further understanding of T_RM_ may help maximize and exploit their potential for therapeutic application and may provide promising avenues for many human disorders.

## Data Availability

Not applicable.

## Abbreviations

T_RM_: Tissue-resident memory T cells
T_EM_: Effector memory T cells
T_CM_: Central memory T cells
T_EF_: Effector T cells
IBD: Inflammatory bowel disease
KLRG1: Killer cell lectin-like receptor G1
MPEC: Memory precursor effector cells
SLEC: Short-lived effector cells
TGF-β: Transforming growth factorβ
S1P: Sphingosine-1-phosphate
S1PR1: Sphingosine-1-phosphate receptor 1
KLF2: Kruppel-like factor 2
VLA-1: Very late antigen-1
CXCL: C-X-C motif chemokine ligand
ICOS: Inducible T-cell co-stimulator
Tfh: T follicular helper cells
CCR: Chemokine receptor
CCL: C-C motif chemokine ligand
Eomes: Eomesodermin
Runx: Runt-related transcription factor
TIL: Tumor-infiltrating lymphocytes
IEL: Intraepithelial lymphocytes
SREBP: Sterol regulatory element-binding protein
FABP: Fatty acid-binding protein
RA: Retinoic acid
MLN: Mesenteric lymph nodes
MHC: Major histocompatibility complex
IFN-γ: Interferon gamma
TNF-α: Tumor necrosis factor alpha
CD: Crohn’s disease
UC: Ulcerative colitis
KO: Knockout
EZH2: Enhancer of zeste homolog 2
ATP: Adenosine triphosphate
ADP: Adenosine diphosphate

## Declarations

### Declarations

Ethics approval and consent to participate.

Not applicable.
